# Enhancing Neuromorphic Robustness via Recurrence Resonance: The Role of Shared Weak Attractors in Quantum Logic Networks

**DOI:** 10.3390/biomimetics11010081

**Published:** 2026-01-19

**Authors:** Yu Huang, Yukio-Pegio Gunji

**Affiliations:** Department of Intermedia Art and Science, School of Fundamental Science and Engineering, Waseda University, Tokyo 169-8555, Japan; yukio@waseda.jp

**Keywords:** recurrence resonance, stochastic recurrent neural network, attractor landscape, local–global interactions, quantum logic

## Abstract

Recurrence resonance, a phenomenon that enhances system computational capability by exploiting noise to amplify hidden attractors, holds significant potential for applications such as edge computing and neuromorphic computing. Although previous studies have extensively explored its characteristics, the underlying mechanism regarding its generation remains unclear. Here, we employed a Stochastic Recurrent Neural Network to simulate neural networks under various coupling conditions. By introducing appropriate inhibitory connections and examining the state transition matrices, we analyzed the characteristics and correlations of attractor landscapes in both global and local systems to elucidate the generative mechanism behind the “Edge of Chaos” dynamics observed under the quantum logic connectivity structure during recurrence resonance. The results show that the strategic introduction of inhibitory connections enriches the system’s attractor landscape without compromising the intensity of recurrence resonance. Furthermore, we find that when neurons are coupled via quantum logic and noise intensity meets specific conditions, the strong attractors of the global system decompose into those of distinct local subsystems, accompanied by the sharing of structurally similar weak attractors. These findings suggest that under quantum logic connectivity, the interaction between the strong attractors of different subsystems is mediated by a background of shared weak attractors, thereby enhancing both the system’s robustness against noise and the diversity of its state evolution.

## 1. Introduction

Distinct from the static processing of classical computers, the brain must continuously adapt to dynamic spatiotemporal inputs arising from a complex environment [1,2,3]. To navigate such diverse stimuli, neural networks in the human brain utilize a hierarchical and modular architecture [4,5,6]. This structural organization facilitates the efficient processing of complex inputs, enabling both robust information integration and signal transmission [7]. Driven by the human brain’s remarkable robustness against environmental perturbations and its superior efficiency in information processing, neuromorphic computing has emerged as a pivotal research frontier [8,9]. Its primary objective is to realize high-parallelism, energy-efficient computational paradigms by mimicking the structural organization and information transmission mechanisms inherent to biological neural systems [10,11]. Within this domain, memristor crossbars have been identified as a premier hardware candidate for physically implementing synaptic architectures and neuronal dynamics, owing to their intrinsic in-memory computing capabilities and analog conductance properties. Despite their broad application prospects such as edge intelligence and physiological signal processing [12], the practical deployment of current memristor-based systems is severely hindered by device non-idealities [13]. These physical limitations induce stochastic errors that significantly degrade inference accuracy and strictly constrain the scalability of large-scale networks. In this manuscript, the term “neuromorphic” is used in a functional and organizational sense rather than as a reference to a specific hardware substrate. The proposed networks are abstract surrogates that capture core neuromorphic principles—such as locality of interaction, recurrence, event-driven state transitions, and robustness to noise—without committing to a particular device physics. This abstraction allows the identified mechanisms to remain substrate-independent, while still being compatible with diverse neuromorphic implementations. In the domain of engineered distributed systems, such structural robustness and fault tolerance are traditionally established through rigorous graph-theoretic frameworks, specifically via diagnosability metrics like g-good-neighbor diagnosability under comparison-based models [14]. Distinct from these static topological guarantees, however, other recent studies suggest that the robustness of this information integration and transmission is closely linked to stochastic perturbations within the neural input [15,16]. Furthermore, noise-driven exploration of the state space has been proposed as a key mechanism for maintaining a balance between functional segregation and integration [17].

Although the precise functional role of noise—often quantified as neural variability—remains a subject of intense investigation, recent frameworks increasingly view it as a fundamental feature for adaptive behavior rather than a mere nuisance [15,18]. The application of noise to enhance both system robustness and computational capacity is well-documented in various domains. Indeed, theoretical and empirical studies have demonstrated that noise can serve as a critical resource, acting as a regularization mechanism to improve generalization in artificial neural networks [19,20] and facilitating phenomena such as stochastic resonance to boost signal processing [16,21]. Beyond the classical stochastic resonance driven by external forcing, there exists a distinct but related phenomenon known as recurrence resonance [22,23]. Unlike stochastic resonance, which requires a periodic external input, recurrence resonance describes the noise-induced exploration of hidden attractors in recurrent neural networks (RNNs).

Indeed, the utility of RNNs as a foundational computational framework for simulating the intrinsic nonlinear dynamics and information processing of biological neural circuits is largely attributed to their structural capacity to generate sustained activity through feedback loops [24,25]. By mimicking the recurrent connectivity ubiquitous in the brain, these networks provide a biologically plausible platform for investigating how the nervous system dynamically stores sequential information and manages internal states [26]. Moreover, the phenomenon of recurrence resonance suggests that the brain may actively modulate internal noise levels to facilitate transitions between dynamical attractors, thereby optimizing cognitive processing [27,28].

In general, the occurrence of recurrence resonance is identified by analyzing the evolutionary trends of entropy and mutual information as a function of increasing noise strength. Specifically, within the low-noise regime, mutual information exhibits a non-monotonic behavior: it initially rises to a distinct peak before rapidly decaying and converging. Crucially, recurrence resonance is confirmed when this peak in mutual information coincides with an intermediate state of entropy—where entropy has increased but has not yet reached its maximum saturation level [23]. Furthermore, this diagnostic framework is scalable. Even in large-scale networks where identifying recurrence resonance via global entropy and mutual information is often challenged by signal dilution or computational constraints, detection remains feasible through local subsystem analysis. Provided the network exhibits a heterogeneous connectivity profile—characterized by the coexistence of dense local clustering and sparse long-range linkages—recurrence resonance can be reliably identified by monitoring the entropy and mutual information of localized sub-modules. This property is termed Local Recurrence Resonance Observability (LRRO) [29]. Notably, under the specific quantum logic connectivity condition [30,31]—a term used here to denote a specific topological structure derived from the algebraic properties of orthomodular lattices, without implying physical quantum effects such as superposition or entanglement—spectral analysis of the neuronal time series—via the Fourier transform—reveals a characteristic 1/f noise profile [29]. This scale-free fluctuation serves as a canonical signature, providing further evidence that the system is operating at the “Edge of Chaos” [32].

The significance of this “Edge of Chaos” regime extends beyond mere spectral characteristics. Theoretically, operating at this critical phase transition is hypothesized to resolve the fundamental trade-off between computational efficiency and universality, enabling systems to maximize information processing capabilities [33,34]. A canonical example is found in elementary cellular automata (ECA), where Rule 110 has been mathematically proven to possess Turing universality, demonstrating that complex computation can emerge from simple rules at the edge of chaos [35]. Another illustrative instance involves asynchronously tuned elementary cellular automata (AT-ECA), where the domain of criticality is broadened through asynchronous updating and locally tuning the consistency between dual modes of transition [36]. Furthermore, research into evolutionary biology and self-organization suggests that such criticality is intimately linked to the emergence of life and complex adaptive behaviors providing a necessary condition for open-ended evolution [37,38,39].

However, translating these broad theoretical advantages of criticality into physical neuromorphic substrates requires a concrete architectural blueprint. Distinct from purely abstract studies, this work focuses on establishing a rigorous link between such dynamical criticality and the physical constraints of hardware implementation. While we do not model specific device physics (e.g., memristive switching dynamics) or quantum phenomena explicitly, we utilize the lattice-based topology as a mathematical abstraction—a structural motif—to design non-local connectivity profiles. This approach allows us to isolate the fundamental mechanism of attractor sharing, thereby providing a theoretical foundation for constructing robust recurrent neuromorphic systems—such as reservoir computing paradigms [40]—that can harness, rather than suppress, the intrinsic noise described earlier. Based on these theoretical foundations, we propose our primary hypothesis: the capacity of the lattice-based quantum logic connectivity structure to generate recurrence resonance and exhibit “Edge of Chaos” characteristics is attributed to a specific mechanism—the facilitation of inter-subsystem information transfer and communication via weak attractors embedded in the background. This architectural mechanism allows subsystems to maintain autonomy and evolve independently, while simultaneously sustaining a requisite level of interaction driven by the continuous modulation of background neuronal connections. A key methodological choice of this study is the deliberate use of small-scale networks as minimal generative systems. Rather than aiming at immediate large-scale performance, our goal is to isolate the structural origin of recurrence resonance and attractor sharing in their simplest nontrivial form. In this sense, the five- and ten-neuron networks should be understood as minimal counterexamples to size-dependent explanations, analogous to the role played by elementary cellular automata in the study of computational universality. The observed phenomena emerge from the relational structure of connectivity and noise modulation, not from fine-tuned parameters or system size.

## 2. Materials and Methods

### 2.1. Stochastic Recurrent Neural Network and Data Analysis

In this study, we adopt a stochastic continuous-valued recurrent neural network framework, which builds upon the deterministic model architecture described in [23]. While the original deterministic model operates purely via hyperbolic tangent dynamics, our approach explicitly incorporates an additive noise term. This allows us to systematically explore how stochasticity modulates the system’s trajectory through its attractor landscape. The dynamics of the network are governed by the continuous internal state variables. Specifically, the temporal evolution of the internal state $u_{i}^{t}$ of neuron *i* is expressed as 

(1) $$u_{i}^{t+1}=\tanh\left(\sum_{j=1}^{n}w_{ij}u_{j}^{t}+r\eta_{i}^{t}\right).$$

where $u_{i}^{t}\in \left[-1,\;1\right]$ represents the continuous state of neuron *i* at time step *t*, corresponding to the ’deterministic’ variable in [23] but subject to stochastic perturbation. $w_{ij}$ denotes the synaptic connection strength between neuron *i* and neuron *j*. The weights are initialized using a truncated normal distribution confined within the deterministic range [p,q]. Specifically, $w_{ij}∼N\left(\mu ,\sigma \right)$ with mean μ=(p+q)/2 and standard deviation σ. These weights are subsequently scaled by the factor *W* to examine the interplay between connection magnitude and noise strength. *n* represents the system size. The noise term $\eta_{i}^{t}$, applied to neuron *i* at time step *t*, is drawn from a standard normal distribution N(0,1), and *r* denotes the noise strength parameter. Although the system evolves in a continuous phase space, we perform a post hoc binarization to analyze the symbolic dynamics, analogous to the output format of standard Boltzmann machines. The discrete state $s_{i}^{t}$ is obtained only after the simulation is complete:

(2) $$s_{i}^{t}=\left\{\begin{array}{cc} 0, & \mathrm{if}\;u_{i}^{t}\leq 0\\ 1, & \mathrm{if}\;u_{i}^{t}>0 \end{array}\right.$$

The state space of the stochastic recurrent neural network at time step *t* is expressed as

(3) $$s^{t}=\left(s_{1}^{t},s_{2}^{t},\ldots ,s_{n}^{t}\right),$$

and the observed symbolic dynamics can be represented as the mapping

(4) $$s^{t}\mapsto s^{t+1}.$$

The two metrics utilized to detect recurrence resonance—entropy and mutual information—are derived from the time series of the observed system’s state (binarized for the calculation of probabilities). The entropy is defined as follows:

(5) $$H\left(X\right)=-\sum_{X}P\left(X\right)\log_{2}P\left(X\right),$$

where *X* is a binary sequence. Mutual information is defined by

(6) $$I\left(X;Y\right)\;=\sum_{X}\sum_{Y}P\left(X,Y\right)\log_{2}\frac{P(X,Y)}{P(X)P(Y)},$$

where both *X* and *Y* are binary sequences. When the observation encompasses the entire system of size *n*, the calculated metrics are designated as global entropy and global mutual information. Conversely, when measurements are restricted to a subsystem of size *m* (where m < n), the resulting metrics are defined as local entropy and local mutual information.

Given the discrete nature of the system states and the presence of noise, we employed State Transition Matrices (STMs) to characterize the distribution of attractors. This approach provides an intuitive visualization of the attractor landscapes across different observation systems. To facilitate matrix construction, we encode each binary sequence into a unique decimal integer $z_{t}$ using the standard binary-to-decimal transformation:

(7) $$z_{t}\;=\sum_{k=1}^{m}s_{k}^{t}2^{k-1},$$

where $z_{t}\;\in \;\left\{0\;,1,\;\ldots ,\;2^{m}\;-\;1\right\}$. Consequently, the indices *x* and *y* in the STM correspond directly to the decimal representations of the system’s state at time *t* and t+1, respectively. Based on this encoding, we construct the STM, denoted as T, with dimensions $2^{m}\;\times \;2^{m}$. Each element $T_{xy}$ represents the conditional probability of the system transitioning from the decimal state *x* to the decimal state *y*:

(8) $$T_{xy}\;=\;P\left(z_{t+1}\;=y\;|\;z_{t}\;=\;x\right)$$

In our numerical analysis, these transition probabilities are estimated empirically from the simulated time series. Let $N_{xy}$ denote the total count of observed transitions from state *x* to state *y* over the entire duration of the simulation. The elements of the transition matrix are computed as

(9) $$T_{xy}\;=\frac{N_{xy}}{\sum_{k=0}^{2^{m}-1}N_{xk}},$$

subject to the normalization condition defined by $\sum_{y}T_{xy}=1$.

Finally, to elucidate the directional information exchange and causal coupling between distinct subsystems, we utilize Transfer Entropy (TE) [41]. Unlike mutual information, which is symmetric and measures static correlations, TE is an asymmetric theoretic-information measure that quantifies the reduction in uncertainty regarding the future state of a target subsystem *B* provided by the knowledge of the past state of a source subsystem *A*, conditioned on *B*’s own history. Let $s_{B}^{t}$ and $s_{A}^{t}$ denote the binary state sequences of the target and source subsystems at time step *t*, respectively. Assuming the system dynamics can be approximated as a first-order Markov process, the TE from subsystem *A* to subsystem *B*, denoted as ${TE}_{A\to B}$, is defined as

(10) $$TE_{A\to B}=\sum_{s_{B}^{t+1},s_{B}^{t},s_{A}^{t}}P\left(s_{B}^{t+1},s_{B}^{t},s_{A}^{t}\right)\log_{2}\frac{P(s_{B}^{t+1}|s_{B}^{t},s_{A}^{t})}{P(s_{B}^{t+1}|s_{B}^{t})},$$

where the summation runs over all possible combinations of states in the binary state space. $P(s_{B}^{t+1},s_{B}^{t},s_{A}^{t})$ is the joint probability distribution, while $P(s_{B}^{t+1}|s_{B}^{t},s_{A}^{t})$ and $P(s_{B}^{t+1}|s_{B}^{t})$ represent the conditional transition probabilities with and without the influence of the source *A*, respectively. A non-zero value of $TE_{A\to B}$ indicates that the past state of subsystem *A* contributes information to the prediction of the current state of subsystem *B* that is not already contained in *B*’s own past, thereby signifying a directed causal influence from *A* to *B*.

Unless otherwise stated, all statistical results are derived from 20 independent trials with different random seeds. Information metrics (entropy and mutual information) are reported as ensemble means; STMs are visualized from a single trial selected randomly at runtime, reflecting the statistically invariant topological structure of the system.

### 2.2. Logical Structure of Neuron Connections

In this study, we specifically investigate the distinct impacts of connectivity weight structures—governed by the diagonal condition and the quantum logic condition—on the system’s attractor landscape. It is crucial to clarify that the term “quantum logic” is used here in its strict order-theoretic sense, referring to the algebraic structure of orthomodular lattices as defined by Birkhoff and von Neumann [42]. It does not imply that the neural network operates on quantum mechanical principles such as superposition or entanglement, nor does it involve quantum hardware. Instead, we utilize this mathematical framework to construct a specific network topology that differs fundamentally from classical random or small-world architectures. Formally, while classical logic corresponds to a Boolean algebra (which is distributive), quantum logic corresponds to an orthomodular lattice (which is non-distributive) [43].

**Figure 1:**
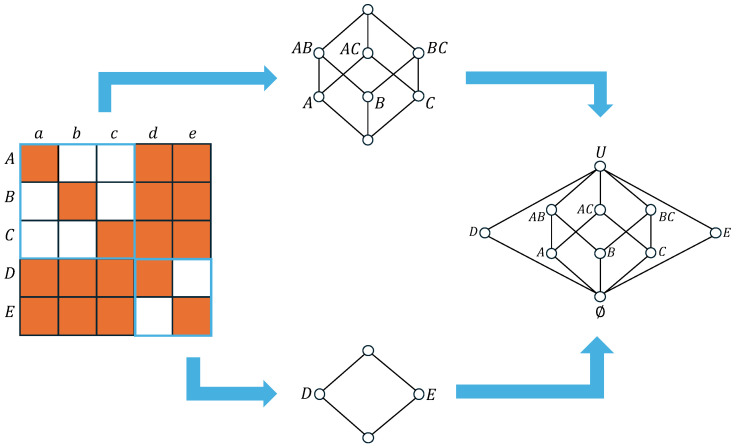
Schematic illustration of the lattice construction from a binary relation. (**Left**) The adjacency matrix representing the binary relation $I\subseteq U_{1}\times U_{2}$ for n=5. Orange cells indicate pairs where the synaptic weight exceeds the threshold ($w_{ij}>\theta$), revealing a block-diagonal structure. (**Center**) The distinct Boolean sub-lattices generated by the independent subsets {A,B,C} (top, $2^{3}$ structure) and {D,E} (bottom, $2^{2}$ structure). (**Right**) The final Hasse diagram representing a non-distributive orthomodular lattice, characteristic of quantum logic. This structure is formed by the “pasting” of the constituent Boolean algebras, which disjoint except for their shared universal bound ∅ and *U*.

To implement this structurally, we employ the concept of “pasting” Boolean subalgebras [44]. Figure 1 provides a schematic illustration of how a binary relation is represented within a lattice. In the context of this study, binary relations are defined with respect to either the connectivity weight structure or the joint probabilities of neuronal states, serving to characterize the specific coupling strength between pairs of neurons. Mathematically, we construct this relation using rough set theory [45,46]. Define two distinct sets of neurons, denoted as $U_{1}=\left\{A,B,C,D,E\right\}$ and $U_{2}=\left\{a,b,c,d,e\right\}$. The state configuration of each set at time step *t* is represented by the vector $s^{t}=\left(s_{1}^{t},s_{2}^{t},s_{3}^{t},s_{4}^{t},s_{5}^{t}\right)$, where the system size is fixed at n=5. A binary relation $I\subseteq U_{1}\times U_{2}$ is derived by determining whether the connection weight $w_{ij}$ exceeds a predefined threshold θ and is expressed as

(11) $$\begin{array}{c} \left(s_{i}^{t},s_{j}^{t}\right)\in I\Leftrightarrow w_{ij}\geq \theta \end{array}$$

(12) $$\begin{array}{c} \left(s_{i}^{t},s_{j}^{t}\right)\notin I\Leftrightarrow w_{ij}<\theta \end{array}$$

In Figure 1, if $(s_{i}^{t},s_{j}^{t})\in I$, the corresponding cell is painted orange; otherwise, it is blank.

The closure operation is defined as follows. For an arbitrary subset $F\subseteq U_{1}$, the closure operation is given by

(13) $$\begin{array}{cc} Cl(F)=R_{*}\left(r^{*}\left(F\right)\right) & \end{array}$$

(14) $$\begin{array}{c} r^{*}\left(F\right)=\{g\in U_{2}|fIg,f\in F\}, \end{array}$$

where fIg represents (f,g)∈I. For any subset $G\subseteq U_{2}$, $R_{*}\left(G\right)$ is expressed as

(15) $$R_{*}\left(G\right)=U_{1}-\left\{f\in U_{1}|fIg,g\notin G\right\}.$$

By direct verification, it can be demonstrated that the set of fixed points of the closure operation constitutes a rough set lattice such as

(16) $$L=\{F\subseteq U_{1}|Cl\left(F\right)=F\}.$$

As each element of a rough set lattice is a set, the order relation between elements is the inclusion relation.

As illustrated in Figure 1, if the relation consists exclusively of diagonal elements, the resulting structure corresponds to a Boolean algebra. Conversely, the presence of diagonal sub-relations bounded by related pairs (f,g)—denoted as fIg—indicates a structure corresponding to the non-distributive orthomodular lattice characteristic of quantum logic. For instance, we observe that Cl({A})={A} and Cl({B,C})={B,C}, whereas Cl({C,D})=U. Consequently, the set {C,D} does not constitute a fixed point of the closure operation. Finally, based on the specified binary relation, we construct the Hasse diagram. This diagram is represented as the union of $2^{3}$- and $2^{2}$-Boolean algebras, which are disjoint except for their shared maximum and minimum elements.

### 2.3. Dynamical Characterization of Attractors

To rigorously classify the dynamical states observed in the STMs, we establish a theoretical framework grounded in the concept of Milnor attractors [47] in this subsection. We distinguish between strong and weak attractors based on their asymptotic stability and measure-theoretic properties and subsequently provide operational criteria for their identification within the discrete context of the stochastic recurrent neural network.

#### 2.3.1. Mathematical Definitions

We first establish a mathematical definition of strong attractors, based on the energy landscape of the system. Let the neural network evolution be conceptualized as a dynamical system on a discrete state space *S*, where the global state at time *t* is denoted by the vector $s^{t}\in {\left\{0,1\right\}}^{N}$. The dynamics of the network are governed by an energy function (or Hamiltonian) E(s), defined as

(17) $$E\left(s\right)=-\frac{1}{2}\sum_{i\neq j}w_{ij}s_{i}s_{j}$$

where $w_{ij}$ represents the synaptic weight between neurons *i* and *j* as before. In this framework, the system’s evolution is viewed as a descent trajectory on this energy landscape, driven by the minimization of E(s) but subject to stochastic fluctuations induced by the noise term $r\eta^{t}$. We define a strong attractor $A_{str}$ as a state that corresponds to a strict local minimum of this energy function. Mathematically, for a state $s^{*}\in A_{str}$, the energy condition $E\left(s^{*}\right)<E\left(s^{\prime }\right)$ holds for all adjacent states $s^{\prime }$ in the Hamming space. Crucially, to qualify as “strong” in the presence of noise, the energy barrier ΔE required to escape the basin of attraction of $s^{*}$ must significantly exceed the effective thermal energy scale determined by the noise strength *r*. Dynamically, this condition results in a state where the self-transition probability significantly exceeds the global average, effectively trapping the system in a stable potential well.

Next, we define the concept of a weak attractor. Adapting Milnor’s formulation to our discrete state space, we characterize a weak attractor $A_{weak}$ as a set whose basin of attraction $B(A_{weak})$ possesses a strictly positive measure (i.e., a non-negligible fraction of the state space), despite the set itself not being asymptotically stable. This definition captures a critical dynamical nuance: while $A_{weak}$ acts as a transient sink for a significant number of system configurations, it inherently possesses escape trajectories (analogous to unstable manifolds in continuous systems) along which stochastic fluctuations can easily induce divergence. In the context of the energy landscape, these sets correspond to shallow metastable valleys or saddles with negligible energy barriers.

#### 2.3.2. Operationalization in STMs

To translate these theoretical definitions into quantitative criteria for our analysis, we leverage the statistical properties derived from the STM constructed in Equation (8). We employ two specific metrics: relative probability gain and diagonal stability. First, to assess the depth of an attractor, we consider its empirical visitation frequency. While the STM elements $T_{xy}$ represent conditional probabilities, the marginal probability $P_{x}$ of observing states *x* can be derived from the transition counts $N_{xy}$:

(18) $$P_{x}=\frac{\sum_{y}N_{xy}}{\sum_{i,j}N_{ij}}$$

We then define the relative probability gain G(x) relative to the uniform baseline $P_{uni}=1/2^{m}$:

(19) $$G\left(x\right)=\frac{P_{x}}{P_{uni}}$$

Second, to quantify the dynamical stability, we directly utilize the diagonal elements of the STM, $T_{xx}$. As defined in Equation (8), $T_{xx}=P\left(z_{t+1}=x|z_{t}=x\right)$ measures the probability of the system remaining in state *x* given it is currently in state *x*.

Based on these metrics, we classify system states into two distinct categories reflecting their stability and visitation profiles. Strong attractors are identified as states exhibiting a substantial probability gain, experimentally characterized by G(x)≫1, coupled with high diagonal stability. In our stochastic system, we identify these strong attractors as states where the self-transition probability $T_{xx}$ significantly exceeds the baseline residence probability (i.e., the average diagonal value of the STM), indicating that the system is effectively trapped in a deep well for extended durations relative to the noise timescale. Conversely, weak attractors are identified as states (or sets of states) that maintain a positive measure above the noise floor (G(x)>1) but possess low diagonal stability comparable to the stochastic baseline ($T_{xx}\ll 1$).

## 3. Results

In this section, we employ a proposed Boltzmann Machine to simulate the evolution of neural networks under varying connectivity conditions. By analyzing the attractor landscape in each scenario, we investigate the specific dynamic behaviors of the system. First, we demonstrate that, while the phenomenon of RR is predominantly governed by network topology rather than inhibitory connections, the introduction of a specific proportion of inhibitory links remains indispensable for suppressing global avalanches and maintaining system stability as the network scale expands. Building on this optimized regime, we further discover that when neuronal connections satisfy quantum logic conditions, strong attractors across subsystems become coherently coupled via shared weak attractors to facilitate information transfer. Notably, this coherence emerges from the global network structure rather than from the precise fine-tuning of synaptic weights.

### 3.1. Impact of Weight Range on Attractor Landscapes Under Consistent RR Trends

**Figure 2:**
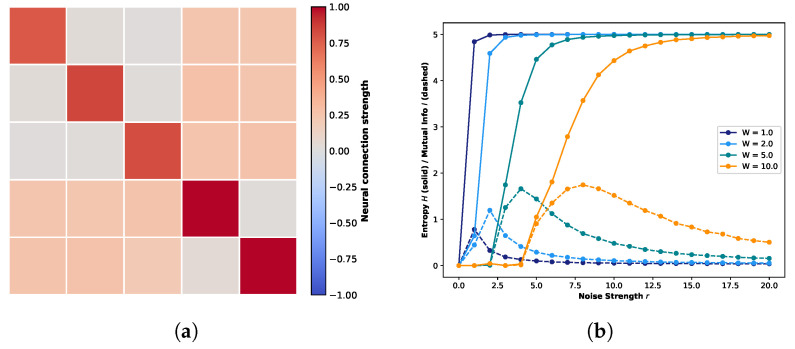
Network connectivity structure and dynamic evolution of information metrics. (**a**) Visualization of the connection weight matrix for the five-neuron network under the quantum logic condition. The color intensity corresponds to the coupling strength: dark red indicates strong connections (0.7–1.0), light orange denotes intermediate connections (0.2–0.3), and gray represents weak or negligible interactions (0.0–0.05). (**b**) The dependence of global entropy (*H*, solid lines) and global mutual information (*I*, dashed lines) on noise strength *r* across different weight scaling factors (*W*) over 20,000 simulation steps. Data points represent the ensemble mean calculated over 20 independent simulation trials. Error bars are omitted for visual clarity as the standard deviation is negligible.

Following the experimental setup of previous studies [29], we initialized the connection weights in the one-dimensional five-neuron network under the quantum logic condition into three distinct ranges: weak (0.0–0.05), intermediate (0.2–0.3), and strong (0.7–1.0). Values within these intervals were sampled from truncated normal distributions defined as N(μ,σ), with μ centered within each range. Based on these settings, we constructed the weight matrix for the five-neuron network under the quantum logic condition, shown in Figure 2a. The connection strengths are color-coded: dark red corresponds to strong coupling, light orange to intermediate coupling, and gray to weak or negligible interactions.

Figure 2b illustrates the evolution of global entropy and global mutual information as a function of noise strength. Different colors correspond to varying connection weight strengths. The solid lines, showing a monotonic increase, represent global entropy, while the dashed lines, which exhibit a rise-and-fall pattern, represent global mutual information. Crucially, the specific regime where global mutual information peaks while global entropy remains below its maximum constitutes the hallmark signature of Recurrence Resonance. The probability P(X) required for these calculations was empirically estimated from the occurrence frequencies over 20,000 simulation steps, where the system state is defined as $X=(s_{1}^{t},s_{2}^{t},s_{3}^{t},s_{4}^{t},s_{5}^{t})$.

**Figure 3:**
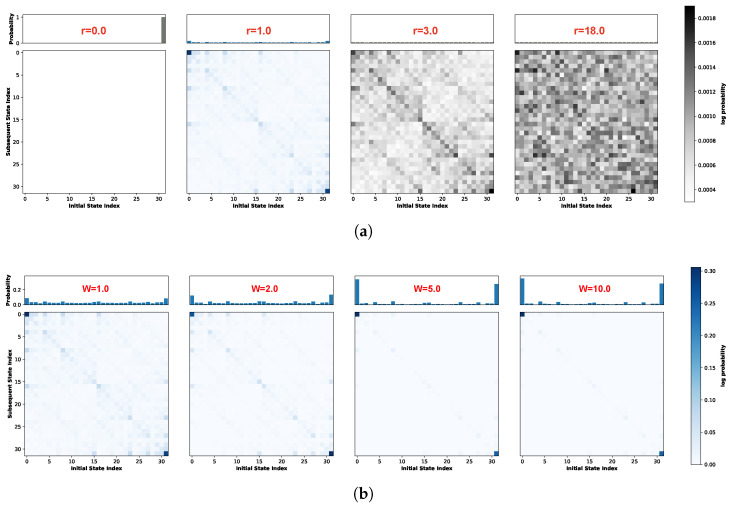
Impact of noise and weight scaling on the system’s state transition dynamics. The State Transition Matrices (STMs) are visualized from a single trial randomly selected at runtime from 20 independent repetitions, illustrating the characteristic topological structure invariant across trials. Each subplot comprises an upper histogram displaying the marginal probability distribution of states and a lower matrix representing transition probabilities. In the matrix, the horizontal axis corresponds to the current state index (ascending left to right), and the vertical axis denotes the subsequent state index (ascending top to bottom). (**a**) STMs at W=1.0 for noise levels r=0.0, 1.0, 3.0 and 18.0, showing the transition from deterministic to stochastic behavior. (**b**) STMs under the Recurrence Resonance (peak mutual information) for weight scaling factors W=1.0, 2.0, 5.0, and 10.0. Higher *W* values significantly enhance the probability of the self-transitions for strong attractors while inhibiting noise effects.

Next, we examine the evolution of the attractor landscape. Figure 3a visualizes the STMs at W=1.0 for four representative noise levels. Each subplot comprises two components: the upper histogram displays the marginal probability distribution, representing the visitation probability of each state during the system’s evolution, while the lower matrix depicts the transition probabilities between states. In this matrix, the horizontal axis corresponds to the current state index (ascending from left to right) and the vertical axis denotes the subsequent state index (ascending from top to bottom). The system transitions from a purely deterministic regime at r=0.0 to a stochastic state dominated by uniform randomness at r=18.0. Crucially, Recurrence Resonance emerges at a noise strength of r=1.0. At this noise level, coinciding with the peak of global mutual information, the STM reveals a hierarchical structure. While States 0 and 31 maintain high self-transition probabilities (indicating strong attractors), the noise induces specific off-diagonal transitions shaped by the network topology. This structure remains partially identifiable at r=3.0 but progressively degrades as stochasticity increases.

Motivated by the potential for dynamic diversity in the global attractor landscape during Recurrence Resonance, we focus our subsequent analysis on the specific noise levels where mutual information is maximized. Figure 3b displays the global STMs for weight scaling factors *W* = 1.0, 2.0, 5.0, and 10.0 under their respective Recurrence Resonance. As the weight scaling factor increases, the coupling between neurons strengthens. This intensification reinforces the stability of strong attractors while rapidly suppressing noise-induced stochastic transitions. As illustrated in the figure, increasing *W* significantly enhances the self-transition probabilities of State 0 and State 31. Concurrently, weak attractors are progressively inhibited; notably, at W=10.0, off-diagonal transitions have virtually vanished, leaving the system dominated by the strong attractors.

However, despite the emergence of Recurrence Resonance, the current connectivity profile limits practical stability. To address this, we next investigate how modulating connection strengths affects the attractor landscape, aiming to balance topological coherence with system stability.

**Figure 4:**
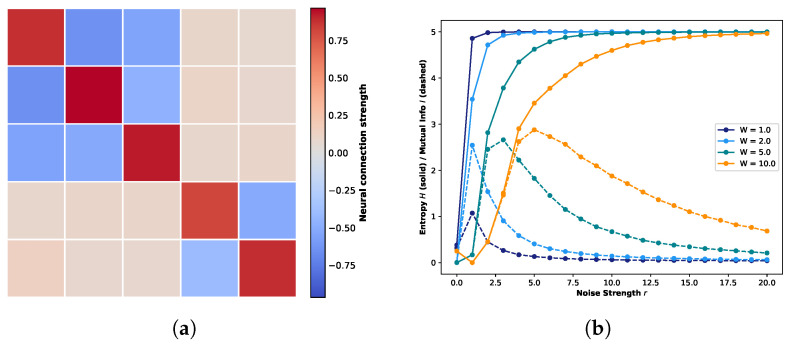
Adjusted connectivity profile and resulting information retrics. (**a**) The tuned weight matrix featuring inhibitory connections (light blue, −0.8–−0.4) and attenuated weak connections (faint orange, 0.0–0.05). Strong excitatory weights (dark red, 0.7–1.0) are preserved. (**b**) Evolution of global entropy (*H*, solid) and mutual information (*I*, dashed) as a function of noise strength *r* for different weight scaling factors *W*, highlighting the regulatory effect of inhibition on system dynamics (*T* = 20,000; ensemble mean, n=20).

Figure 4a presents the weight matrix for the five-neuron one-dimensional network, preserving the topological structure introduced in Figure 2 but with modified connection strengths. In this configuration, the originally negligible interactions (gray regions) are reassigned as inhibitory connections, with weights sampled from a truncated normal distribution ranging from −0.8 to −0.4 (visualized as light blue regions). Concurrently, the previously intermediate connections are attenuated to a weaker range of 0.0 to 0.05 (appearing as faint orange colors). Crucially, the strong excitatory connections along the diagonal (dark red) are maintained within their original range of 0.7 to 1.0 to preserve the primary signal pathways. Under this modified connectivity profile, Figure 4b illustrates the resulting trends of global entropy and global mutual information. Consistent with Figure 2, different colors represent distinct weight scaling factors, while solid and dashed lines denote entropy and mutual information, respectively. As illustrated in the figure, the introduction of inhibitory connections leads to a marked elevation in the global mutual information peaks compared to the purely excitatory network in Figure 2. Notably, these peaks emerge at lower noise intensities. A particularly striking amplification is observed at W=2.0, where the peak value exhibits a more than twofold increase compared to W=1.0. This phenomenon is mechanistically attributable to the regulatory role of inhibitory connections. Unlike the purely excitatory regime, which drives the system toward rapid saturation, the presence of inhibition introduces a dynamic interplay of enhancement and suppression. This E/I balance prevents the system from prematurely converging to fixed points, thereby enhancing the temporal correlation between consecutive states and extending the duration of transient dynamics. Consequently, the system exhibits a richer repertoire of state evolutions.

**Figure 5:**
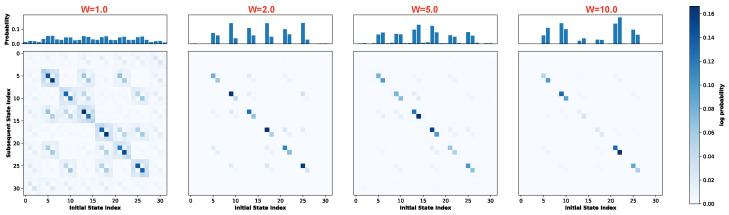
State Transition Matrices (STMs) under the adjusted connectivity profile (randomly selected trial, n=20). The matrices visualize the global state transitions at the Recurrence Resonance peak (maximum mutual information) for weight scaling factors W∈{1.0, 2.0, 5.0, 10.0}. Strong attractors here are distributed more uniformly along the diagonal. At lower weights (W=1.0), the off-diagonal regions are densely populated by weak attractor structures, reflecting increased state diversity. As *W* increases to 10.0, these off-diagonal transitions are effectively suppressed, isolating the dominant strong attractors.

Building on the aforementioned adjustments, Figure 5 illustrates the system’s global STMs at the point of Recurrence Resonance across various weight scaling factors *W*. Consistent with the findings in Figure 3b, an increase in *W* further enhances the self-transition probabilities of strong attractors while reducing the overall prevalence of weak attractors. However, distinct differences emerge under the current configuration. First, regardless of the weight scaling factor *W*, there is a substantial increase in the total number of strong attractors. Second, these strong attractors are no longer confined to the extreme states (top left and bottom right corners); instead, they are distributed more uniformly along the diagonal. Furthermore, particularly at W=1.0 where the global connection strength remains unamplified, the off-diagonal regions are densely populated by weak attractor structures similar to the diagonal components. This observed landscape corroborates our earlier conclusion: the introduction of inhibitory connections significantly diversifies the system’s intrinsic state evolution.

The comparative analysis of the five-neuron network under identical quantum logic conditions reveals a critical insight: far from diminishing the intensity of Recurrence Resonance, the strategic introduction of inhibitory connections actually diversifies the system’s global attractor landscape. To further validate this conclusion, we extend our analysis to a network of equivalent size subject to diagonal conditions.

**Figure 6:**
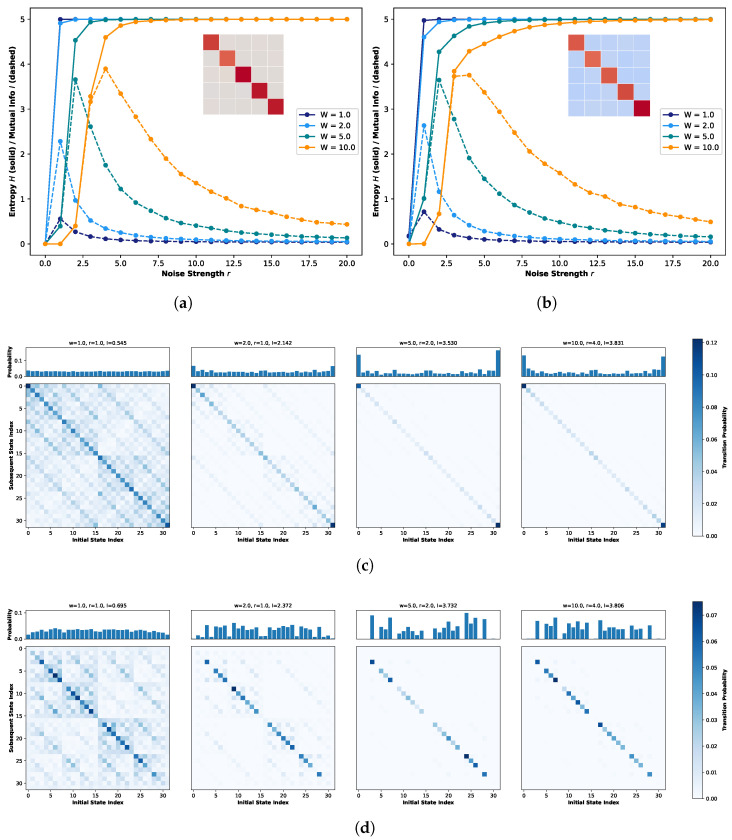
Comparative analysis of dynamics in purely excitatory versus inhibitory-modulated diagonal networks. (**a**,**b**) Evolution of global entropy (*H*, solid lines) and mutual information (*I*, dashed lines) as a function of noise strength *r* (T=20,000; ensemble mean, n=20). (**a**) Represents the purely excitatory network (inset: off-white negligible off-diagonals, 0.0–0.05), while (**b**) introduces inhibitory connections (inset: light blue off-diagonals, −0.26–−0.16). Both maintain strong excitatory diagonals (dark red, 0.7–1.0). Note that the peak trends of mutual information remain consistent across weight scaling factors (*W*) in both configurations. (**c**,**d**) The corresponding State Transition Matrices (STMs) captured at the Recurrence Resonance peak (randomly selected trial, n=20). (**c**) The purely excitatory system exhibits state transitions heavily skewed towards extreme states (0 and 31). In contrast, (**d**) the inhibitory-modulated system reveals a more diversified attractor landscape with sharper boundaries and increased transition probabilities between extreme states, as indicated by the marginal probability distributions (top histograms).

Figure 6a,b illustrates the five-neuron one-dimensional networks based on a diagonal topology, featuring purely excitatory connections and the introduction of inhibitory connections to off-diagonal components, respectively. In the weight matrices, dark red indicates strong coupling (0.7–1.0), off-white represents negligible coupling (0.0–0.05), and light blue denotes inhibitory connections (−0.26–−0.16), with all values sampled from their respective truncated probability distributions.

Figure 6a clearly demonstrates that under the purely excitatory regime, Recurrence Resonance is robustly observed across varying weight scaling factors. Due to the tight self-coupling of individual neurons, self-reinforcement effects drive a rapid surge in the peak of global mutual information as *W* increases. Conversely, in Figure 6b, despite the introduction of inhibitory connections and a slight saturation in peak growth at W=10.0, the peak magnitudes and overall trajectories of global mutual information remain remarkably consistent with those of the purely excitatory system across all tested scaling factors.

However, this macroscopic consistency does not extend to the global attractor landscape. Figure 6c,d depicts the global STMs observed at the Recurrence Resonance peak for the purely excitatory and inhibitory-modulated systems, respectively. While both exhibit a generally similar distribution of strong and weak attractors, the system with inhibitory connections displays a more distinct definition of strong attractors with sharper boundaries separating them from weak attractors. Furthermore, an examination of the marginal probability distributions reveals a key divergence: In the purely excitatory case, state evolution aligns with quantum logic conditions, skewing heavily towards the extreme states (0 and 31). In contrast, the system with inhibitory connections exhibits more active state transitions between these extremes. This distinction corroborates our earlier conclusion: the strategic introduction of inhibitory connections fosters the diversification of system state evolution, enriching the dynamic repertoire.

Taken together, the analysis of the five-neuron one-dimensional network indicates that although Recurrence Resonance is fundamentally structurally driven, we demonstrate that the appropriate inclusion of inhibitory connections, far from abolishing Recurrence Resonance, acts as a critical regulatory mechanism.

### 3.2. Interconnectivity of Strong Attractors via Weak Attractors Under Quantum Logic

Building on the conclusions of the preceding subsection, we proceed to investigate the specific impact of the quantum logic connectivity structure on the attractor landscapes within distinct subspaces, incorporating the estabished inhibitory modulation. By comparative analysis with control experiments and utilizing Transfer Entropy (TE) to visualize the directional flow of information, we aim to elucidate how this specific topology faciliates communication between subspaces. Specifically, we demonstrate that the quantum logic structure enables inter-subspace information transfer mediated by weak attractors embedded in the background dynamics.

**Figure 7:**
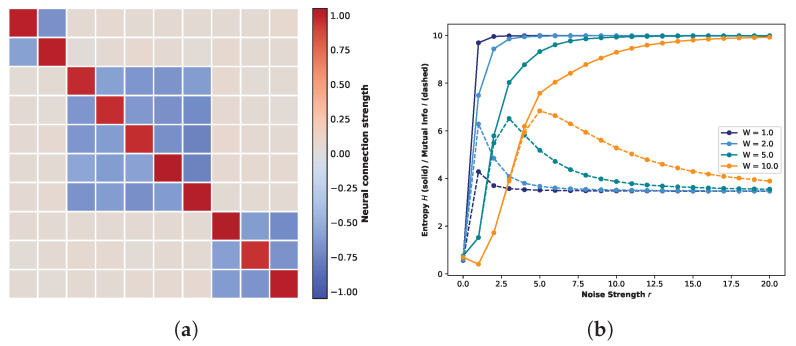
Connectivity structure and information dynamics of the ten-neuron network under quantum logic conditions. (**a**) Visualization of the weight matrix constructed as a disjoint union of $2^{2}\text{-},\;2^{5}\text{-},\;\mathrm{and}\;2^{3}\text{-}$Boolean algebras sharing common greatest and least elements. Color coding denotes connection strengths: strong excitatory (dark red, 0.95–1.05), inhibitory (light blue, −0.75–−0.55), and negligible (off-white, 0.02–0.08). (**b**) Global entropy (*H*, solid lines) and mutual information (*I*, dashed lines) as a function of noise strength *r* across varying weight scaling factors *W*. To ensure adequate sampling of the extended state space, the simulation duration was extended to 100,000 steps (ensemble mean, n=20). Note that due to the immense state space size, mutual information maintains an elevated baseline (≈3.6) yet still exhibits distinct Recurrence Resonance peaks in the low-noise regime.

Figure 7a presents the weight matrix of a ten-neuron one-dimensional neural network with a connectivity structure governed by the quantum logic condition. Specifically, the topology corresponds to the disjoint union of $2^{2}$-, $2^{5}$- and $2^{3}$-Boolean algebras, sharing common greatest and least elements. In the matrix, deep red entries denote strong coupling ranging from 0.95 to 1.05, off-white entries signify negligible interactions (0.02–0.08), and light blue regions represent inhibitory connections with weights between −0.75 and −0.55. All values sampled from their respective truncated probability distributions.

**Figure 8:**
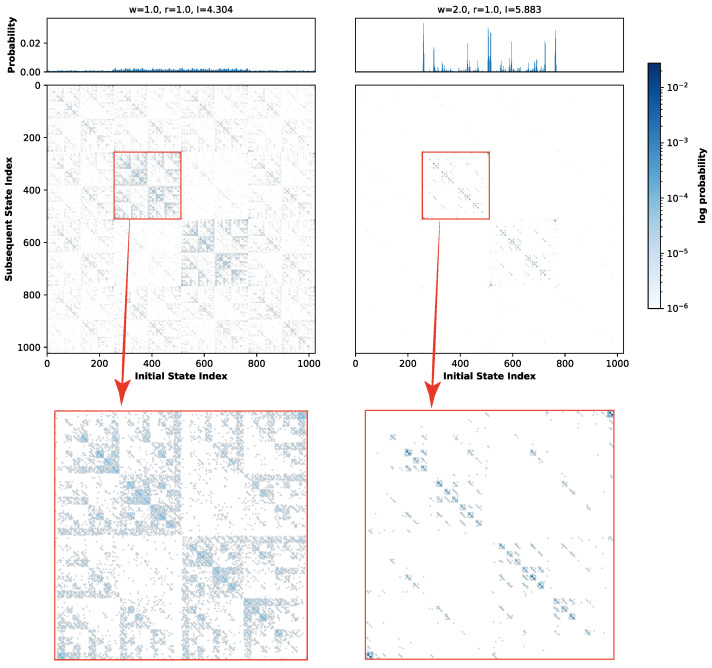
Noise-induced ordering and structural crystallization in the global attractor landscape. The figure compares the State Transition Matrices (randomly selected trial, n=20) and marginal probability distributions at the Recurrence Resonance peak (r=1.0) for weight scaling factors W=1.0 (left column) and W=2.0 (right column). The STMs are plotted on a logarithmic scale to enhance the visibility of weak transition pathways, while the upper marginal probability histograms remain on a linear scale. The bottom panels present zoomed-in insets of the off-diagonal regions highlighted by the red bounding boxes (x,y∈[250, 500]). (**Left Panel** W=1.0): The system exhibits a diffuse, fractal-like structure. The zoomed-in inset reveals self-similar geometric patterns within the transition probabilities, visually characterizing the metastable weak attractors that facilitate state space exploration. The marginal probabilities are uniformly low, indicating that despite the presence of resonance, the dynamics remain largely stochastic with weak attractor definition. (**Right Panel** W=2.0): A doubling of the connection strength triggers a transition to ordered dynamics. The inset shows that the fractal noise is effectively suppressed, and the system crystallizes into distinct block-diagonal clusters. This is accompanied by the emergence of sharp peaks in the marginal probability distribution, confirming the formation of robust strong attractors and the segregation of noise.

Figure 7b illustrates the evolution of global entropy and global mutual information as a function of noise strength under this specific weight matrix. Here, solid lines denote global entropy, while dashed lines represent global mutual information. Given the expanded state space associated with the ten-neuron architecture, a simulation duration of 20,000 steps is insufficient for adequate state exploration. Consequently, to ensure statistical convergence while maintaining computational feasibility, the simulation period was extended to 100,000 time steps. Distinct colors correspond to the four different weight scaling factors *W*. As observed in the figure, despite extending the simulation to 100,000 time steps, the global mutual information does not converge to 0 but remains elevated at a baseline of approximately 3.6, attributable to the immense magnitude of the system’s state space. Nevertheless, a distinct peak in global mutual information is still evident within the low-noise-strength regime, followed by a gradual decay, confirming the successful observation of Recurrence Resonance. Consistent with the trends observed in the five-neuron network, the peak magnitude increases with the weight scaling factor *W*. Notably, a substantial enhancement is observed primarily when transitioning from W=1.0 to W=2.0; for higher values (W=5.0 and W=10.0), the peak intensities exhibit a saturation effect with negligible variation compared to W=2.0. Consequently, the subsequent analysis of local subspaces will focus specifically on the distinct regimes of W=1.0 and W=2.0. Figure 8 presents the global STMs for the ten-neuron one-dimensional neural network governed by the aforementioned connectivity structure. The left and right panels illustrate the global state transition dynamics for weight scaling factors W=1.0 and W=2.0, repectively. Due to the expansive magnitude of the global state space, the empirical visitation probability for individual states remains relatively low throughout the 100,000-step evolution, fluctuating around an order of magnitude of $10^{-2}$. Consequently, to enhance the visual contrast of transition intensities, a logarithmic scale was applied to the values in the STMs. In contrast, the marginal probability distributions presented in the upper panels remain plotted on a linear scale.

As indicated in Figure 7b, at W=1.0, although Recurrence Resonance is observable, the magnitude of the peak shows only a modest elevation, implying that stochasticity remains the prevailing force in the system. This characteristic is reflected in the STM shown in the left panel. The marginal probability distribution (top histogram) reveals that the visitation probability for any global state remains uniformly low; while states in the central region exhibit slightly higher probabilities compared to the extremes, the overall state evolution is dominated by randomness. However, despite this stochastic dominance, the STM reveals a distinct, regular fractal structure. The darker coloration along the central diagonal indicates a higher propensity for transitions into these regions. Furthermore, the diagonal is surrounded by weak attractor structures that mirror the diagonal’s intensity pattern, exhibiting a self-similar characteristic where coupling strength decays from the diagonal outwards.

The right panel of Figure 8 vividly exemplifies the phenomenon of noise-induced ordering characteristic of Recurrence Resonance. As anticipated from the W=2.0 curve in Figure 7b, doubling the global connection strength precipitates a marked transformation in the marginal probability distribution. The distribution shifts from a diffuse profile to one characterized by sharp peaks concentrated in the central regions, while the peripheral states are notably suppressed compared to the W=1.0 case. This ordering is visually manifested in the STM: the diffuse fractal structure observed previously effectively vanishes. Instead, the dynamics crystallize into distinct, high-probability clusters along the central diagonal. Within these regions, the self-transitions are significantly reinforced, while the off-diagonal weak attractors are filtered out, leaving only faint residual traces aligned with the primary diagonal.

**Figure 9:**
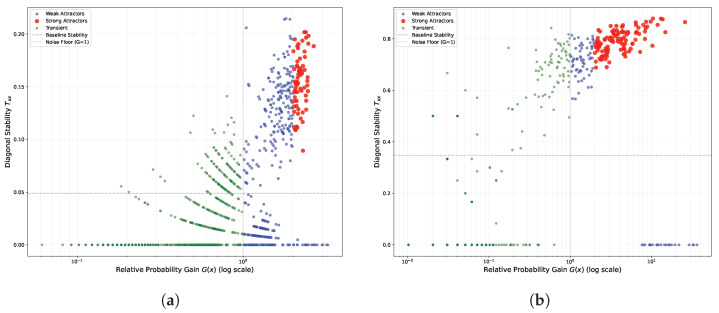
Quantitative classification of the attractors in the ten-neuron network under the quantum logic condition. The scatter plots map the diagonal stability ($T_{xx}$) against the relative probability gain (G(x), on a logarithmic scale) for all system states, identifying distinct dynamical roles: strong attractors (red), weak attractors (blue), and transient noise (green). The vertical dotted line represents the theoretical noise floor (G(x)=1), while the horizontal dashed line indicates the empirical baseline stability derived from the STM. (**a**) At W=1.0, the system exhibits the gap between stochastic noise and stable states. (**b**) At W=2.0, a topological phase transition is observed; the dynamics crystallize into a distinct cluster of high-stability strong attractors (upper-right), while the weak attractor population undergoes a functional differentiation. Specifically, a ’structural hub’ class emerges that is strictly distinct from the diffusive background, whereas the high-stability weak attractors remain proximal to the noise floor, creating a selective gating interface.

To elucidate the distinct dynamical regimes underlying the observed STMs, we performed a quantitative classification of attractors based on the relative probability gain, G(x), and diagonal stability, $T_{xx}$. Figure 9 presents the resulting attractor classification map for the 10-neuron network subject to the quantum logic connectivity profile (cf. Figure 7). Figure 9a,b illustrates the system’s state distribution under weight scaling factors of W=1.0 and W=2.0, respectively. In these plots, states are color-coded according to their dynamical roles: strong attractors (red), weak attractors (blue), and transient noise (green). The horizontal dashed gray line represents the empirical baseline stability, while the vertical dotted gray line denotes the theoretical noise floor (G(x)=1).

Consistent with the operational definitions established in Section 2.3.2 and accounting for the dimensionality of the global state space ($2^{10}=1024$), we adopted a hierarchical classification strategy. Specifically, any state falling at or below the uniform randomness threshold (G(x)≤1.0) is strictly categorized as transient noise. For states exceeding this noise floor, we apply a rigorous dual criterion to identify strong attractors: these states must exhibit both substantial visitation frequency, defined by a threshold of G(x)>2.0, and high diagonal stability, exceeding 1.5 times the baseline value ($T_{xx}>1.5\times \;\mathrm{baseline}$). Consequently, all remaining states that satisfy the visibility condition (G(x)>1.0) but fail to meet these elevated thresholds for strong attractors are classified as weak attractors.

Under the W=1.0 regime, we first examine the distribution of strong attractors (red). These states represent the system’s most frequently visited configurations with prolonged residence times. While their relative probability gain exceeds the uniform randomness benchmark ($P_{uni}\approx 0.00098$) by a factor of two, the absolute visitation frequency oscillates around 0.2%. However, considering the high dimensionality of the state space and the significant noise intensity (r=1.0), this magnitude constitutes a statistically significant deviation from chance, strictly adhering to our operational definition. Spatially, these strong attractors cluster near the classification threshold rather than separating into a distinct, isolated group. This continuum suggests that at W=1.0, although the system achieves peak recurrence resonance, the dynamics remain heavily influenced by stochasticity, preventing the complete “crystallization” of attractors. Conversely, the region to the left of the noise floor (G(x)<1) is dominated by transient noise (green), which accounts for the vast majority of the state space. These states exhibit either negligible visitation frequencies or insufficient stability, indicating that their occurrence is driven primarily by stochastic fluctuations rather than intrinsic structural dynamics. Occupying the intermediate regime between transient noise and strong attractors are the weak attractors (blue), which constitute the metastable background corresponding to the faint, fractal-like structures observed in the STM. A critical inspection of the scatter plot reveals that this category bifurcates into two distinct dynamical roles based on their stability profiles. The first subset consists of high-stability, low-gain states located above the stability baseline (≈0.05). These states possess local potential barriers comparable to those of strong attractors but are associated with small basins of attraction; this implies that while they are stochastically difficult to access, they function as deep local traps once entered. In contrast, the second subset comprises low-stability, high-gain states positioned below the stability baseline. These states function primarily as “structural hubs”: although they lack the potential depth to retain the system for extended periods, their high relative gain (G(x)>1) indicates that they are topologically central and frequently traversed. Together, these complementary weak attractor types facilitate the system’s global connectivity without enforcing rigid locking.

In marked contrast, the landscape under the W=2.0 regime (Figure 9b) exhibits a fundamental topological reorganization. The strong attractors (red) are no longer clustered near the classification threshold (G(x)≈2); instead, they migrate significantly towards the upper-right quadrant, forming a dispersed yet distinct high-probability cluster. Quantitatively, these states achieve a mean diagonal stability of approximately 0.8, with relative probability gains reaching five times the uniform baseline, indicating the formation of deep, robust potential wells. Concurrently, the classification of weak attractors population undergoes a functional differentiation, eliminating the gradient-like transition observed at W=1.0. Specifically, the population bifurcates into two distinct sub-classes: a structural hub group that is strictly distinct from the diffusive background, and a high-stability cluster bordering the stochastic baseline (G(x)≈1), which creates a selective gating interface. A similar dichotomy emerges within the transient noise regime (green): these states segregate into a high-stability cluster proximal to the noise floor and a diffusive low-stability component approaching $T_{xx}\approx 0$. Corroborating the STM analysis, we attribute this crystallized distribution to the global amplification of synaptic weights. This intensification effectively suppresses noise-driven stochasticity, enforcing a rigid structural hierarchy that underpins the maximization of mutual information observed in the system.

**Figure 10:**
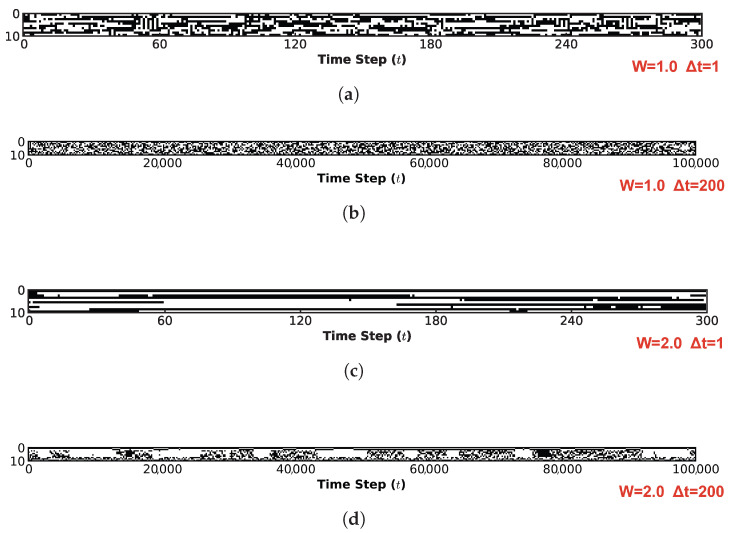
Microscopic and macroscopic views of system evolution. (**a**,**c**) Short-term evolution (Δt=1) for (**a**) W=1.0 and (**c**) W=2.0. While W=1.0 shows noise-corrupted trajectories, W=2.0 demonstrates stable propagation assisted by noise-induced ordering. (**b**,**d**) Long-term evolution (Δt=200) over 100,000 steps. (**b**) At W=1.0, dynamics are indistinguishable from random noise. (**d**) At W=2.0, the system exhibits intermittency, where fine-grained clusters persist within a quiescent background, indicating robust information preservation at the critical transition point.

Figure 10 visualizes the system’s spatiotemporal evolution, demonstrating how the global state transition characteristics identified in Figure 8 are manifested in the temporal domain. This figure characterizes the spatiotemporal evolution of the system through two distinct lenses: microscopic and macroscopic. Across all four subplots, the horizontal axis presents the time evolution steps, while the vertical axis denotes the neuron index. Figure 10a,c illustrates the microscopic mechanisms of information flow under W=1.0 and W=2.0, respectively. For this perspective, a high-resolution sampling interval of 1 step was employed over an initial observation window of 300 steps. In contrast, Figure 10b,d depicts the macroscopic steady-state behaviors for W=1.0 and W=2.0. To evaluate the sustainability of information propagation within the system, the complete evolutionary trajectory of 100,000 steps was monitored using a coarse-grained sampling interval of 200 steps.

Comparing Figure 10a and Figure 10c corroborates our earlier analysis: at W=1.0, stochastic noise remains the dominant driver. Although the system exhibits a fractal-like organization with identifiable attractor distributions, the differentiation between strong and weak attractors is indistinct. In the spatiotemporal domain (Figure 10a), this is manifested as continuous trajectories (black lines) heavily punctuated by scattered noise artifacts (black dots), indicating that the system’s state biases towards chaos, thereby compromising the integrity of information preservation. In stark contrast, at W=2.0 (Figure 10c), noise effectively functions as a stabilizing force for ordered dynamics. The spatiotemporal trajectories appear coherent and clean, with virtually no interstitial noise artifacts, ensuring stable information transmission. Crucially, the presence of noise prevents the system from converging into a rigid, frozen state; instead, it induces spontaneous yet stable switching between different metastable states. From a macroscopic vantage point, the dynamics in (Figure 10b) exhibit negligible regularity, resembling pure stochastic noise. Conversely, in (Figure 10d), the system largely settles into a quiescent state (indicated by the disappearance of extensive black regions) after the inital transient. However, critical information is preserved and propagated through localized, persistent clusters (fine trajectories). This behavior strongly suggests that under the specific quantum logic connectivity and appropriate noise levels, the system operates at the “Edge of Chaos”—maintaining a delicate balance where evolutionary order coexists with the diversity of state dynamics.

**Figure 11:**
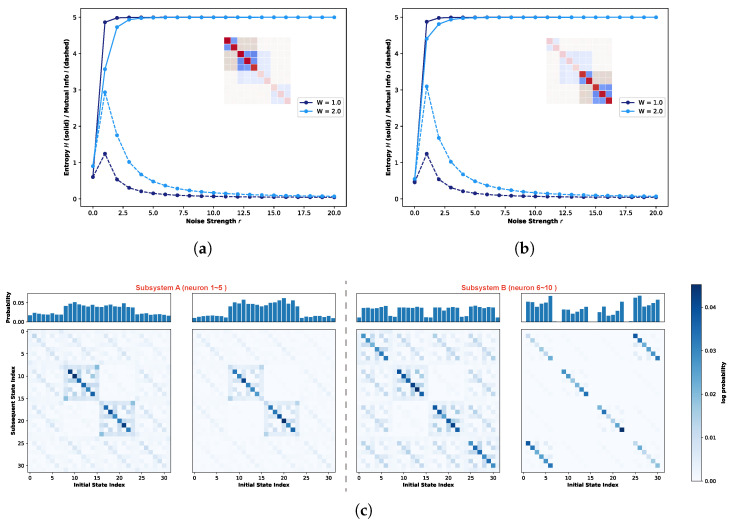
Comparative analysis of information dynamics and distinct attractor landscapes in partitioned subspaces. (**a**,**b**) Local entropy and mutual information curves (ensemble mean, n=20) for (**a**) Subspace A (neurons 1–5) and (**b**) Subspace B (neurons 6–10), showing consistent quantitative behaviors. (**c**) Comparison of STMs (randomly selected trial, n=20) at the resonance peak (visualized on a linear scale). Subspace A (left) exhibits a centralized attractor structure, whereas Subspace B (right) displays a diagonal alignment. In both cases, increasing the weight factor from W=1.0 to W=2.0 significantly enhances the contrast between strong and weak attractors, as quantified by the marginal probability distributions.

Subsequently, we analyze the subspace dynamics of the ten-neuron one-dimensional neural network based on the connectivity matrix presented in Figure 7a. To facilitate a comparative analysis, the system is partitioned into two distinct subspaces: Subspace A (comprising neurons 1–5) and Subspace B (comprising neurons 6–10). Figure 11a,b illustrates the evolution of local entropy and local mutual information as a function of noise strength for Subspace A and Subspace B, respectively. Given that the state space of each subspace is significantly reduced ($2^{5}=32$ states), a simulation duration of 20,000 steps is sufficient to ensure ergodic sampling, in contrast to the 100,000 steps required for the global system. Consequently, the simulation was set to 20,000 steps. Guided by the global mutual information trends, this analysis focuses specifically on the regimes of W=1.0 and W=2.0. Figure 11c visualizes the STMs and the corresponding marginal probability distributions for both subspaces at the Recurrence Resonance peak. Notably, unlike the global analysis, these local representations are plotted on a linear scale.

Initially, a comparative analysis of Figure 11a,b reveals that the local entropy and, in particular, the local mutual information for both distinct subspaces exhibit nearly identical evolutionary trends. Moreover, the peak magnitudes of mutual information under both W=1.0 and W=2.0 conditions remain remarkably consistent. However, as visualized in Figure 11c, despite sharing identical internal connectivity structures and comparable Recurrence Resonance intensities, the STMs of the two subspaces unveil drastically divergent attractor landscapes. The panel to the left of the vertical dashed line illustrates the landscape for Subspace A. At W=1.0, the strong attractor structure is predominantly localized within the central region of the state space. Specifically, the diagonal components within this central square region exhibit a deeper blue coloration, indicating a higher transition probability towards these states. Simultaneously, weak attractors oriented along the diagonal are dispersed throughout the background surrounding this central cluster. Upon increasing the weight scaling factor to W=2.0, while the saturation of the strong attractors deepens slightly, the suppression of weak attractors is far more pronounced. The faint background attractors are effectively filtered out. This shift is quantified by the marginal probability distributions: the visitation probability for central states increases marginally, whereas the probability for peripheral weak attractors is reduced by approximately 50%. Conversely, the panel to the right depicts Subspace B. In distinct contrast to Subspace A, the strong attractors in Subspace B are aligned almost perfectly along the main diagonal of the state space, although the background shares a similar distribution of weak attractors. At W=2.0, these background components virtually vanish, leaving only residual, relatively strong attractors in the upper-right and lower-left corners.

To elucidate the specific role of the quantum logic-governed connectivity structure in facilitating information transfer between subsystems, we conducted a series of comparative control experiments. Specifically, we analyzed two modified topological configurations: a diagonal condition, where all off-diagonal background connections were completely eliminated, and a perturbed configuration, where the quantum logic condition was deliberately violated by selectively blocking a portion of the background connections.

**Figure 12:**
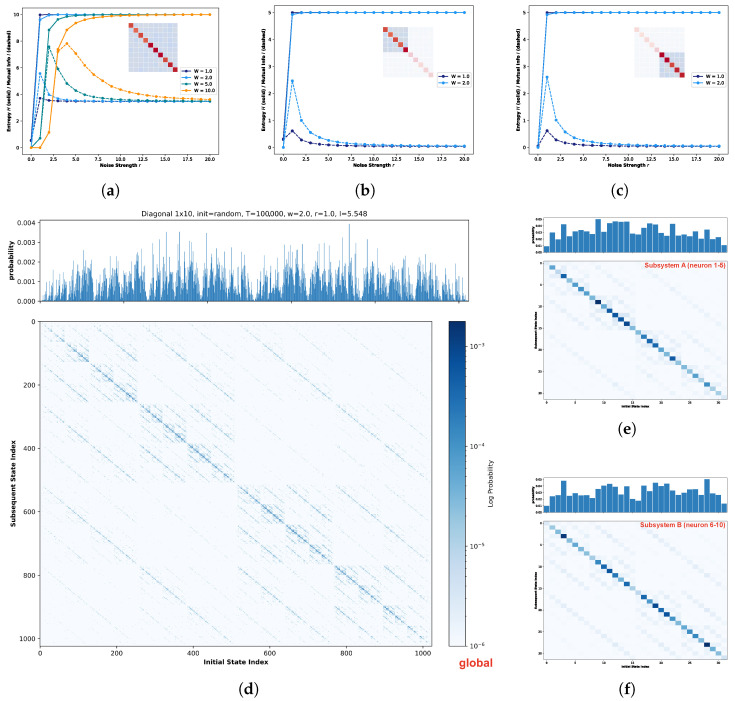
System dynamics and attractor landscapes under the strictly diagonal control condition. (**a**–**c**) Dependence of entropy (*H*, solid lines) and mutual information (*I*, dashed lines) on noise strength (ensemble mean, n=20). (**a**) Global system response for weight scaling factors W=1.0, 2.0, 5.0, and 10.0. (**b**,**c**) Local responses for Subsystem A (neurons 1–5) and Subsystem B (neurons 6–10) at W=1.0 and 2.0. Note the identical evolutionary trends observed in both subsystems, contrasting with the divergent behaviors in the quantum logic case. (**d**–**f**) State Transition Matrices (STMs) and marginal probability distributions at the recurrence resonance (W=2.0; randomly selected trial, n=20). (**d**) Global STM plotted on a logarithmic scale. Strong attractors are distributed strictly along the main diagonal, reflecting a dominance of self-convergence behavior. (**e**,**f**) Local STMs for Subsystems A and B plotted on a linear scale. Both subsystems exhibit an irregular probability distribution with diagonal strong attractors. The trivial structural similarity across global and local scales (scale invariance) implies that the system evolution is driven by the independent stochastic dynamics of individual neurons, devoid of structured inter-subsystem communication.

Figure 12 illustrates the impact of noise strength on the state evolution of the global system and its subsystems for a ten-neuron network configured with an exclusively diagonal connectivity matrix. Figure 12a depicts the variations in global entropy and global mutual information as a function of increasing noise strength. Consistent with previous analyses, the four distinct colors correspond to weight scaling factors of W=1.0, 2.0, 5.0, and 10.0. Figure 12b,c presents the corresponding local entropy and mutual information trends for Subsystem A (neurons 1–5) and Subsystem B (neurons 6–10), respectively. To maintain methodological consistency with the previous quantum logic experiments, the simulation durations were kept invariant: 100,000 steps for the global system and 20,000 steps for the subsystems. Observation of Figure 12a reveals that, similarly to the quantum logic case, the global mutual information does not converge to zero. This is attributed to the simulation duration being insufficient to fully sample the expanded state space. Nevertheless, the overall trajectory clearly exhibits the characteristic signatures of Recurrence Resonance. Furthermore, given the strictly diagonal topology where self-coupling dominates the connectivity profile, the peak magnitude of global mutual information escalates rapidly with the increasing weight scaling factor *W*. Regarding Subsystems A and B, we restricted our analysis to the W=1.0 and W=2.0 regimes to ensure comparability. Inspection of Figure 12b,c indicates that both subsystems display nearly identical evolutionary trends in local entropy and mutual information. Notably, at W=2.0, the role of noise in enhancing system state stability becomes particularly pronounced. Consequently, the subsequent analysis of STMs will focus primarily on the W=2.0 scenario.

Figure 12d–f presents the marginal probability distributions of state visitation and the corresponding STMs for the global system, Subsystem A, and Subsystem B, respectively. As depicted in Figure 12d, the global STM under the diagonal condition exhibits a fractal structure reminiscent of the quantum logic condition. However, a critical distinction exists: strong attractors are strictly distributed along the main diagonal of the entire state space, maintaining high structural consistency with the surrounding weak attractors. This implies that under this connectivity profile, the state evolution of individual neurons is heavily biased towards self-convergence. Given the high degree of neuronal independence, the global system dynamics tend to be driven by stochastic fluctuations rather than structured, coordinated evolution. The local analyses of Subspaces A and B further corroborate this interpretation. Inspection of the marginal probability distributions reveals a noteble contrast to the clustered organization observed under the quantum logic condition; here, strong and weak attractors display an irregular, quasi-random distribution. Furthermore, the STMs of both subsystems are not only identical to each other but also exhibit a trivial similarity to the global matrix. This scale invariance indicates that regardless of the observation scale (global or local), state evolution is governed by independent dynamics, with no evidence of specific communication or information exchange between distinct partitions.

**Figure 13:**
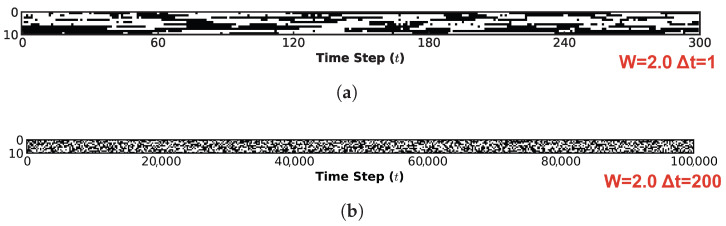
Spatiotemporal instability under the diagonal condition at W=2.0. (**a**) Microscopic view (Δt=1). Although some information flow is visible, the trajectories are short-lived and heavily corrupted by noise compared to the quantum logic case, reflecting poor stability. (**b**) Macroscopic view (Δt=200). The long-term evolution is characterized by a complete absence of structural patterns. The system is dominated by random fluctuations (noise saturation), confirming that the diagonal topology cannot sustain the orderly yet diverse dynamics required for critical computation.

The spatiotemporal evolution dynamics of the system provide further corroboration of these observations. Figure 13 illustrates the spatiotemporal evolution of the ten-neuron network under the diagonal condition at W=2.0. Analogously to the analysis in Figure 10, we examine the system from both microscopic and macroscopic perspectives. Figure 13a depicts the microscopic evolution with a sampling interval of Δt=1 over the intial 300 steps. In distinct contrast to Figure 10c, although stable evolutionary trajectories (black lines) are discernible, a significant proportion of noise-induced stochastic transitions (scattered black dots) persists. Moreover, the continuity of these stable trajectories is notably shorter compared to the quantum logic configuration under the same weight scaling factor. This indicates that at the microscopic level, while information flow occurs, both the fidelity of information transmission and the stability of the system states are significantly compromised compared to the quantum logic case. Figure 13b further elucidates this instability from a macroscopic vantage point (sampling interval Δt=200, total duration 100,000 steps). Here, the system appears entirely saturated with stochastic noise artifacts, lacking any coherent structure and exhibiting characteristics of purely random evolution. This serves as a compelling counter-example that reinforces our previous conclusion: the quantum logic connectivity structure uniquely positions the system at the “Edge of Chaos”, effectively balancing state diversity with evolutionary order.

**Figure 14:**
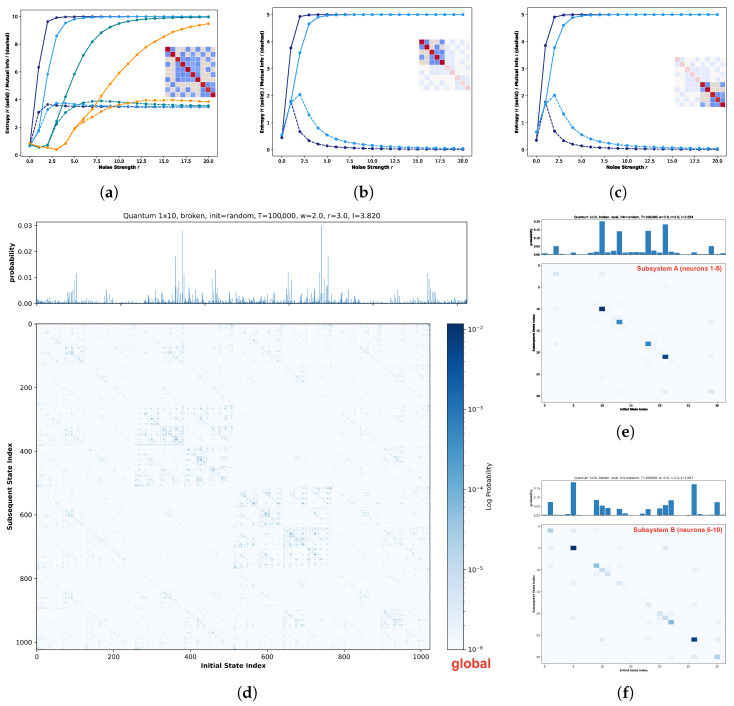
System dynamics and attractor landscape degradation under the randomized background topology (Control Experiment 2). (**a**–**c**) Dependence of entropy (*H*, solid lines) and mutual information (*I*, dashed lines) on noise strength (ensemble mean, n=20). (**a**) Global system response. In contrast to the intact quantum logic condition, the mutual information peak is markedly suppressed, indicating that noise fails to facilitate coherent global ordering when the background topology is disrupted. (**b**,**c**) Local responses for Subsystem A and B (W=1.0, 2.0), where resonance signatures persist locally despite global incoherence. (**d**–**f**) State Transition Matrices (STMs) and marginal probability distributions at W=2.0 (randomly selected trial, n=20). (**d**) Global STM plotted on a logarithmic scale. The randomization of background connections leads to a degradation of the fractal self-similarity observed in Figure 8. This is accompanied by a flattening of the marginal probability distribution, reflecting an increased prevalence of intermediate-strength attractors. (**e**,**f**) Local STMs for Subsystems A and B plotted on a linear scale. Both subsystems exhibit a tendency towards diagonal clustering, signifying the loss of the structural complementarity (centralized vs. diagonal) and background coherence essential to the quantum logic configuration.

Figure 14 presents the results of another control experiment, designed to further verify the specificity of the quantum logic condition. In this setup, rather than completely eliminating the off-diagonal background connections (as in the diagonal condition), the specific topological structure of the background was deliberately disrupted while preserving connectivity density. Following the established analytical format, Figure 14a–c illustrates the dependence of global and local entropy/mutual information on noise strength. Experimental parameters remain consistent with previous settings: Subsystem A (neurons 1–5), Subsystem B (neurons 6–10), and weight scaling factors W=1.0 and 2.0. Simulation durations were maintained at 100,000 steps for the global system and 20,000 steps for the subsystems. Figure 14a reveals a critical divergence: upon disrupting the coherent quantum logic background structure, the global mutual information response is markedly suppressed. Unlike the distinct resonance peaks observed in the quantum logic and diagonal conditions, the global system here exhibits a negligible peak, implying that under this randomized connectivity profile, noise fails to facilitate coherent global ordering. Conversely, inspection of Figure 14b,c indicates that the subsystems retain distinct resonance signatures, with mutual information displaying observable peaks. Furthermore, the evolutionary trends of entropy and mutual information are nearly identical across both subsystems. However, a notable difference from the previous conditions is the attenuated sensitivity to the weight scaling factor; the impact of increasing *W* on the system dynamics is less pronounced in this perturbed configuration.

Figure 14d–f presents the marginal probability distributions of state visitation and the corresponding STMs for the global system and the two subsystems, respectively. A comparative analysis of Figure 14d against the quantum logic condition (Figure 8, right panel) reveals a significant structural degradation: upon disrupting the specific quantum logic background topology while maintaining the same weight scaling factor, the propagation of weak attractors within the global state space markedly increases. Although the central region retains traces of the original quantum logic pattern, the global landscape no longer exhibits a distinct fractal structure. Concurrently, the marginal probability distribution indicates a reduction in dominant strong attractors accompanied by a proliferation of intermediate-strength states. This flattening of the probability landscape implies a diminished intensity gradient among attractors, suggesting that stochasticity plays a more prominent role in the system’s actual evolution. The local analyses in Figure 14e,f further highlight the loss of structural coordination. While the STMs of the two subsystems exhibit some differences, a crucial deviation from the original quantum logic condition is observed: strong attractors in both subsystems tend to cluster along the diagonal, lacking the distinct structural complementarity seen previously (where Subspace A was centralized and Subspace B was diagonal). Furthermore, unlike the quantum logic case (Figure 11) where subsystems shared a coherent background weak attractor pattern, the background patterns here are disjointed. This contrast underscores that the intact quantum logic topology is essential for fostering structural complementarity and global coherence across subsystems.

**Figure 15:**
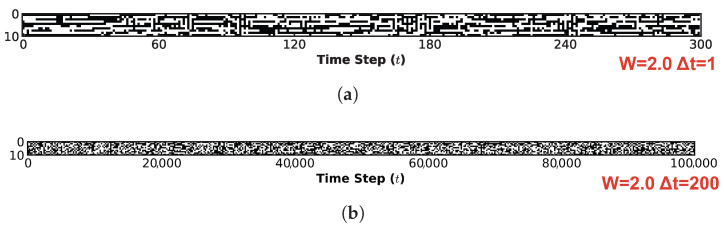
Loss of coherent evolution under the broken quantum logic condition. (**a**) Microscopic view (Δt=1). The system displays severe instability. Stable trajectories are notably shorter and more disrupted by noise than in the diagonal control case, reflecting a breakdown in information perservation. (**b**) Macroscopic view (Δt=200). The system exhibits purely chaotic dynamics with no emergent structure. The saturation of stochastic noise confirms that disrupting the precise quantum logic weights eliminates the system’s ability to maintain the delicate balance between order and diversity.

Figure 15 illustrates the global spatiotemporal evolution of the system under the disrupted quantum logic condition. Figure 15a presents the microscopic perspective, maintaining the same sampling interval and observation window as in previous analyses. It it evident that while segments of stable evolution (black lines) persist, they resemble the diagonal condition in their transience, being frequently disrupted by noise. Indeed, the instability observed here appears even more pronounced than in the diagonal case. Figure 15b depicts the macroscopic perspective, utilizing a coarse-grained sampling interval of 200 steps over the entire evolutionary course. From this global vantage point, the information flow fails to manifest any coherent spatiotemporal patterns, instead exhibiting characteristics of pervasive stochasticity. This further underscores that even a partial disruption of the quantum logic connectivity structure is sufficient to precipitate a descent into a chaotic state, effectively eliminating the system’s capacity for ordered evolution.

**Figure 16:**
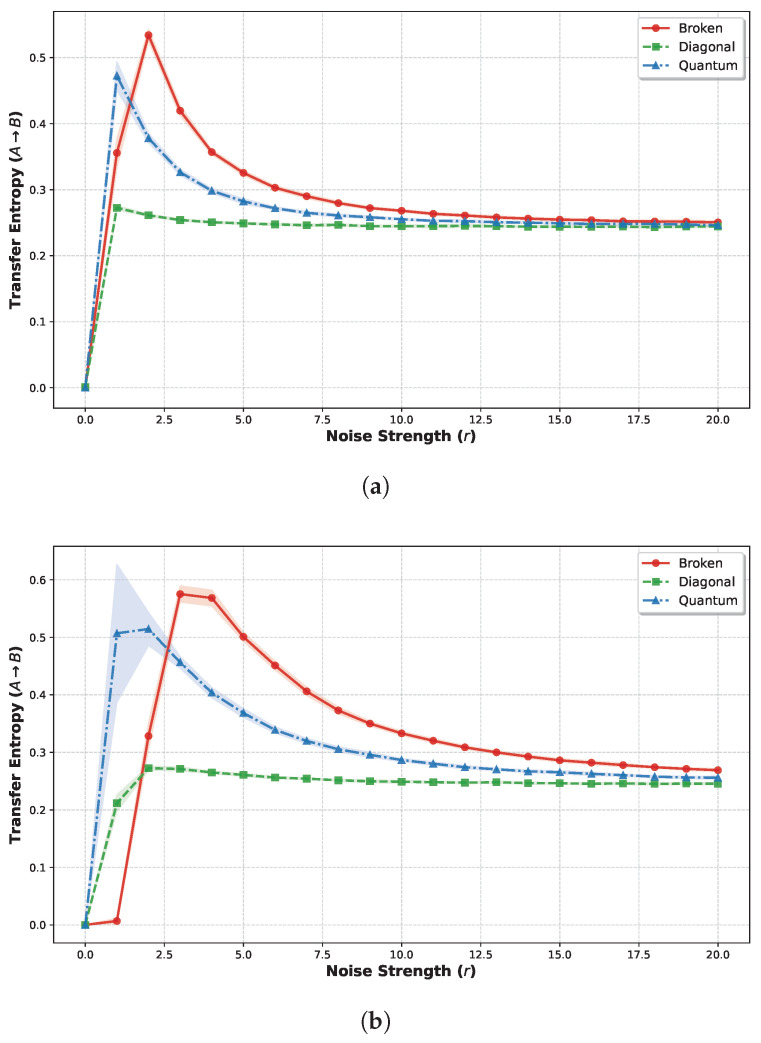
Quantification of directed information flow via Transfer Entropy ($TE_{A\to B}$) as a function of noise strength. The transfer entropy from subsystem A to subsystem B is plotted for three distinct connectivity architectures: broken quantum logic (red), diagonal (green), and quantum logic (blue). (**a**) Results for weight scaling factor W=1.0. (**b**) Results for weight scaling factor W=2.0. In both panels, lines and markers represent the ensemble mean calculated over 20 independent simulation trials. The shaded error bands explicitly represent the uncertainty, defined as the mean ± one standard deviation (SD). Note that while the broken quantum logic condition exhibits a higher peak magnitude, the quantum logic condition displays significantly wider error bands (particularly at W=2.0), quantitatively reflecting the high trial-to-trial variability and dynamic metastability associated with this regime.

Finally, we employ TE to strictly quantify the directional information flow and interaction dynamics between subspaces under varying connectivity architectures. Figure 16 illustrates the TE from Subsystem A (neurons 1–5) to Subsystem B (neurons 6–10) as a function of noise strength, comparing three distinct weight structures: the quantum logic condition, the diagonal condition, and the broken quantum logic condition. The results corresponding to weight scaling factors W=1.0 and W=2.0 are presented in Figure 16a,b, respectively. In both panels, the horizontal axis denotes the noise strength, while the vertical axis represents the TE flowing from subsystem A to subsystem B. Each data point corresponds to the ensemble mean calculated over 20 independent simulation trials for a given noise strength. The shaded regions accompanying each curve illustrate the variability of the results, representing the range of the mean ± one standard deviation (SD).

Across both weight scaling regimes (W=1.0 and W=2.0), the $TE_{A\to B}$ trajectories exhibit qualitatively similar trends that correlate strongly with the mutual information profiles. Under W=1.0, the broken quantum logic condition demonstrates the highest peak magnitude (0.5341±0.0086 bits, mean ± SD), followed by the quantum logic condition (0.4724±0.0214 bits) and the significantly lower diagonal condition (0.2726±0.0032 bits). Amplifying the weight scaling factor to W=2.0 increased these magnitudes while preserving the hierarchical order: the peak TE rose to 0.5752±0.0140 bits for the broken quantum logic condition and 0.5145±0.0285 bits for the quantum logic condition, whereas the diagonal condition remained comparatively suppressed (0.2726±0.0034 bits). Statistical analysis (One-way ANOVA) confirms significant differences across connectivity conditions (p<0.001). Specifically, independent *t*-tests revealed the difference in peak TE between the broken quantum logic and quantum logic conditions is significant (*t*-test, p<0.01). Furthermore, the shaded regions in Figure 16, representing the standard deviation σ across 20 independent trials, quantify the uncertainty of these estimates. Notably, the quantum logic condition exhibits a significantly wider error band at its peak compared to the broken quantum logic condition, quantitatively supporting our claim of higher dynamic variability in the quantum regime.

## 4. Discussion

### 4.1. Relation to Neuromorphic Substrates

The abstract neuron and synapse models employed here admit natural mappings to existing neuromorphic substrates. For example, the stochastic update dynamics can be interpreted as rate-based or event-driven spiking neural networks, while the heterogeneous and inhibitory couplings correspond to effective synaptic plasticity realized in memristive or mixed-signal circuits. From this perspective, the reported robustness does not rely on a specific hardware realization, but instead reflects a class of architectural principles implementable across spiking neural networks, memristive crossbars, and reservoir computing frameworks. Therefore, the contribution of this work lies not in proposing a new device-level implementation, but in identifying a dynamical and topological mechanism that explains how neuromorphic robustness can emerge despite intrinsic noise and device variability.

### 4.2. Structural Mechanisms of Recurrence Resonance and Criticality

Our results demonstrate that the introduction of appropriate inhibitory connections enables the system to significantly enrich its attractor landscape while preserving the magnitude of recurrence resonance (i.e., maintaining high mutual information intensity). Moreover, compared to other connectivity profiles, the quantum logic condition establishes a unique dynamic balance: subsystems maintain autonomy in their evolution, yet achieve effective communication through background weak attractors. We posit that this specific interaction mode is the primary driver positioning the system at the “Edge of Chaos”. Crucially, this finding fills a critical gap in the literature by elucidating the structural generative mechanism of recurrence resonance, distinguishing our architectural approach from purely phenomenological observations.

In this study, we first demonstrated the pivotal role of inhibitory connections in enriching the dynamical behaviors of neural networks. Specifically, when employing the weight distributions based on the quantum logic or the diagonal conditions from prior research, we observed that despite the presence of sparse connectivity (negligible interactions), the restriction to an exclusively excitatory regime renders the system highly susceptible to converging into trivial states characterized by sustained quiescence or global saturation (Figure 3 and Figure 6). Such dynamical instabilities are known to precipitate pathological phenomena, including unregulated neuronal avalanches or epileptiform synchronization [48]. By strategically introducing inhibitory connections while preserving the underlying topological architecture, we observed that strong attractors became more uniformly distributed across the state space, effectively shifting away from the polarized extreme states. This redistribution not only enhances dynamical stability but also significantly enriches the diversity of state evolution. These findings align with prior research positing that recurrence resonance augments system computational capacity by unmasking and exploiting hidden attractors within the landscape [29].

Second, building upon the premise of inhibitory modulation, we conducted an in-depth analysis of the inter-subsystem connectivity specific to the quantum logic condition. The construction of the STM for the global system revealed that the attractor landscape under this condition exhibits a distinct fractal structure (Figure 8). Characteristic of a multi-stable system, this topology implies that the state space is partitioned into a series of nested metastable states. In this regime, the system resides within a localized sub-region for a duration before stochastic noise induces a transition to an adjacent region. It is precisely this hierarchical architecture that enables the system to reconcile two competing dynamical requirements: the preservation of local stability and the capacity for global exploration. In the context of computational neuroscience, such local stability is analogous to short-term memory retention, while the macroscopic traversal of the state space corresponds to long-term memory integration. From the perspective of nonlinear dynamics, this fractal organization serves as a hallmark of a system operating at the “Edge of Chaos” [32]. As comparative benchmarks, we also computed the STMs for the diagonal and broken quantum logic conditions (Figure 12d and Figure 14d). While traces of fractal structure are discernible in these controls, a quantitative assessment reveals a significant degradation in terms of scaling properties and attractor density compared to the quantum logic condition. This implies that although these systems retain a marginal capacity for stability and exploration, their ability to sustain and balance these functions is severely compromised. This disparity is further elucidated by the spatiotemporal evolution patterns shown in Figure 10, Figure 13 and Figure 15. Microscopically, while individual neuronal information is preserved to some degree in the control conditions, the retention duration is markedly shorter than in the quantum logic case; furthermore, instability in interaction dynamics prevents the maintenance of global coherence. Macroscopically, with the exception of the quantum logic condition, the global evolution is indistinguishable from random noise, reflecting an inherent structural instability.

Complementing the global analysis, we also computed the STMs for the two subsystems under each connectivity architecture (Figure 11c, Figure 12e,f and Figure 14e,f). In the quantum logic condition, the two subsystems exhibit divergent landscapes of strong attractors, yet strikingly, they share a nearly identical pattern of background weak attractors. Crucially, a superposition of these distinct subsystem landscapes reconstructs a structure that is structurally congruent with the global attractor landscape. Conversely, in the diagonal condition, the STMs of the two subsystems are virtually identical to each other and bear a trivial resemblance to the global pattern. This stark constrast suggests that under the quantum logic regime, global state evolution is not merely a sum of independent parts but is jointly archestrated by the two subsystems. This implies the existence of active inter-subsystem communication, which is likely mediated by the shared network of background weak attractors. The broken quantum logic condition serves as an intermediate case. While the distribution of strong attractors in its subsystems is not identical (unlike the diagonal case), their combination fails to preserve the structural integrity of the global strong attractors. Furthermore, the distribution of background weak attractors becomes inconsistent. This indicates that even minor topological perturbations to the quantum logic structure rupture the coherent link between local and global dynamics.

Finally, to quantify the information transfer between subsystems across different conditions, we calculated the Transfer Entropy (TE). Compared to the diagonal condition, the quantum logic condition exhibits a significant directional influence from Subsystem A to Subsystem B; specifically, the current state of A exerts a substantial predictive impact on the future evolution of B. Although the broken quantum logic condition manifests an even higher TE magnitude, corroboration with spatiotemporal evolution plots suggests this is largely an artifact of the system’s chaotic dynamics. When Subsystem B resides in a high-entropy state, its future evolution is poorly predicted by its own history. In this context, despite the partitioning of the system, Subsystem A continues to act on B, and any external input from A exerts a statistically amplified effect due to B’s intrinsic unpredictability. Furthermore, the peak $TE_{A\to B}$ in the broken quantum logic condition occurs at a strictly higher noise strength compared to both the quantum logic and diagonal conditions. This indicates that, given equivalent weight strengths, the broken architecture requires a stronger stochastic driving force to access this high-TE regime. This observation resolves an apparent paradox regarding the magnitude of information flow versus its functional utility. While the broken quantum logic condition yields a higher peak TE, we interpret this not as superior communication, but as noise-driven information flow. In this regime, the target subsystem exhibits maximal entropy, rendering it hyper-sensitive to external perturbations. Consequently, the high TE reflects the transmission of stochastic noise rather than structured signals. In contrast, the quantum logic condition maintains a moderate TE magnitude but exhibits significant trial-to-trial variability (indicated by the extensive shaded error bands). This dynamic variability suggests that the system is not rigidly locked into a high-flow or low-flow state but operates at a critical point of metastability, switching transiently between distinct dynamical regimes. Functionally, this is advantageous because it prevents the system from being overwhelmed by global noise while allowing for state-dependent signal integration. Thus, the quantum logic architecture optimizes the structure and controllability of information flow, rather than merely maximizing its raw magnitude, behavior that further corroborates the system’s operation at the “Edge of Chaos”.

Recent advancements in neuromorphic computing have shifted the paradigm from strictly suppressing hardware noise to actively exploiting it. The intrinsic stochasticity of emerging devices, such as cycle-to-cycle variability in memristors, can serve as a valuable computational resource for stochastic computing and Bayesian inference [49]. However, while these existing approaches predominantly focus on leveraging stochasticity at the device level, our work introduces a complementary mechanism at the architectural level. Complementary to this, from a hardware architecture perspective, while current memristor arrays are predominantly fabricated as dense crossbars, state-of-the-art design methodologies (e.g., structured pruning [50,51] and tiling [52,53]) increasingly advocate for introducing logical sparsity to optimize performance. Our dynamical analysis reveals that the quantum logic topology does far more than simply reduce connection density. It fundamentally reshapes the system’s dynamical landscape. Under this topology, the network transcends simple metastability (solely strong attractors). Instead, it generates a sophisticated coexistence of strong attractors and weak attractors that function as structural hubs. These weak attractors effectively channel the intrinsic noise to drive transitions between distinct functional states. This implies that rather than striving to precisely control the binary switching of every non-ideal memristor, we can harness their collective stochasticity—guided by the weak attractors—to orchestrate the self-organized switching dynamics of the system state, thereby establishing a robust physical substrate for reservoir computing.

A limitation of the current study is the reliance on a small-scale simulation. We acknowledge that strictly verifying scale-invariant properties, such as 1/f noise, is statistically challenging in finite-size systems due to boundary effects and limited spectral resolution. However, we deliberately frame the present results as a “motif analysis”. The ten-neuron network serves as the minimal functional unit capable of explicitly demonstrating the interplay between strong local attractors and shared weak attractors. By focusing on this minimal motif, we isolate the fundamental dynamical mechanisms that might be obscured by the complexity of larger state spaces. Theoretically, the proposed quantum logic topology possesses intrinsic scalability. The construction method, based on the pasting of Boolean subalgebras, allows for modular network expansion by iteratively fusing additional Boolean blocks, analogous to crystal lattice growth. From a computational perspective, analyzing recurrence resonance in large-scale versions of these networks remains feasible. While the global state space grows exponentially, rendering global entropy and mutual information calculations computationally prohibitive, our diagnostic framework relies on LRRO. As demonstrated in previous studies [29], this property enables the detection of global resonance regimes via the monitoring of local subsystems. Consequently, the analytical cost scales linearly with the number of modules (i.e., linearly with network size) rather than exponentially with network size. We therefore posit that the dynamical roles of attractors identified in this minimal motif provide a robust foundation for generalizing to larger, modularly constructed neuromorphic architectures.

Although this study has demonstrated that the quantum logic connectivity architecture facilitates inter-subsystem information transfer via background weak attractors—thereby exhibiting characteristics akin to Self-Organized Criticality (SOC)—and has provided mechanistic insights into the qualitative and quantitative emergence of 1/f noise during recurrence resonance, we have not yet definitively mapped the specific evolutionary trajectories of the system state. Consequently, future research will prioritize analyzing the evolution of attractors as a primary entry point to delineate the operational characteristics of this weight structure. Furthermore, we aim to generalize this framework to quantum logic structure naturally derived from Bayesian and Inverse Bayesian inference, assessing the universality of these findings across broader contexts. Ultimately, this line of inquiry seeks to establish a novel paradigm for enhancing the stability and universality of neuromorphic computing. We acknowledge that systematic scaling analyses will be necessary to quantitatively characterize how these structures evolve in larger networks. However, the existence of the mechanism itself—namely, the mediation of inter-subsystem interactions by shared weak attractors—can already be demonstrated at minimal scales. Establishing this logical origin is a prerequisite for any subsequent large-scale or hardware-oriented implementation.

## 5. Conclusions

Motivated by the objective of leveraging noise to enhance computational performance in neural networks, this study investigates the potential of implementing a specific structural mechanism within the system. Through the strategic introduction of inhibitory connections, we successfully facilitated the emergence of recurrence resonance while effectively averting the pathological extremes of global saturation and stochastic disorder. Crucially, our results demonstrate that inhibition is indispensable for fostering system diversity: the strategic introduction of inhibitory connections actually enriches the global attractor landscape. By precluding the polarization of extreme states, inhibition cultivates a broader dynamic repertoire, thereby enabling the system to sustain high information capacity without becoming entrapped in trivial fixed points.

Furthermore, the specific topological structure of quantum logic instantiates a unique mode of “weak” coupling. This architecture engenders a hierarchical organization: strong attractors guarantee the stability of local subsystems, whereas shared “weak attractors” facilitate effective inter-subsystem communication. This structural coherence stands in sharp contrast to the control conditions analyzed: the strict diagonal topology results in the functional isolation of subsystems, while the randomized topology leads to the disintegration of coherent structures. Consequently, the intact quantum logic lattice is identified as the critical determinant that permits global integration while preserving local autonomy.

Ultimately, the system epitomizes the hallmark characteristics of the “Edge of Chaos”, effectively balancing the ordered retention of information with flexible state transitions. This equilibrium is empirically substantiated by the optimization of Transfer Entropy at a specific noise strength, a phenomenon driven by the interplay between topological architecture and stochastic fluctuations. These insights suggest that the mathematical formalism of quantum logic lattices offers a robust blueprint for designing neuromorphic systems capable of self-organized criticality. Future work will aim to scale this framework to larger networks and explore its concrete implementation as a practical computational neural model. 

## Data Availability

The data presented in this study are available on request from the corresponding author.
